# The Vestigial Esterase Domain of Haemagglutinin of H5N1 Avian Influenza A Virus: Antigenicity and Contribution to Viral Pathogenesis

**DOI:** 10.3390/vaccines6030053

**Published:** 2018-08-10

**Authors:** Zhiqiang Zheng, Subha Sankar Paul, Xiaobing Mo, Yu-Ren Adam Yuan, Yee-Joo Tan

**Affiliations:** 1 Department of Microbiology and Immunology, Yong Loo Lin School of Medicine, National University Health System (NUHS), National University of Singapore, Singapore 117545, Singapore; miczz@nus.edu.sg (Z.Z.); subhasankarpaul@u.nus.edu (S.S.P.); 2 Department of Biological Sciences, Faculty of Science, National University of Singapore, Singapore117558, Singapore; mox@nus.edu.sg (X.M.); dbsyya@nus.edu.sg (Y.-R.A.Y.); 3 National University of Singapore (Suzhou) Research Institute, Suzhou Industrial Park, Suzhou 215123, Jiangsu, China; 4 Institute of Molecular and Cell Biology, A*STAR (Agency for Science, Technology and Research), Singapore 138673, Singapore

**Keywords:** influenza, neutralising antibodies, vestigial esterase, antibody dependent cell-mediated cytotoxicity, pH-induced conformational changes

## Abstract

Initial attempts to develop monoclonal antibodies as therapeutics to resolve influenza infections focused mainly on searching for antibodies with the potential to neutralise the virus in vitro with classical haemagglutination inhibition and microneutralisation assays. This led to the identification of many antibodies that bind to the head domain of haemagglutinin (HA), which generally have potent neutralisation capabilities that block viral entry or viral membrane fusion. However, this class of antibodies has a narrow breadth of protection in that they are usually strain-specific. This led to the emphasis on stalk-targeting antibodies, which are able to bind a broad range of viral targets that span across different influenza subtypes. Recently, a third class of antibodies targeting the vestigial esterase (VE) domain have been characterised. In this review, we describe the key features of neutralising VE-targeting antibodies and compare them with head- and stalk-class antibodies.

## 1. Introduction

Being the major surface glycoprotein present on the envelope of the influenza A virus (IAV), many studies have been devoted to understanding the structure of HA and its antigenicity. HA is a type 1 transmembrane protein that is assembled as a homotrimer in the endoplasmic reticulum and transported to the plasma membrane via the secretory pathway. HA is further cleaved into HA1 and HA2 by a protease provided by the host system. The two subunits remain linked by a disulphide bridge [1]. Structurally, each subunit consists of a membrane-proximal helix-rich stem structure primarily composed of HA2 with some HA1 residues, and a membrane-distal receptor-binding globular domain comprised of HA1 [2].

In terms of antigenicity, antibodies against HA are mainly responsible for protection via vaccination [3,4]. With advances in monoclonal antibody (mAb) technologies, large numbers of neutralising mAbs have been generated and their characterisation yields insights into the antigenicity and functionality of epitopes/motifs in HA. In addition, passive immunotherapy utilising broadly neutralising mAbs has been proposed as a promising treatment, and there has been extensive characterisation of these mAbs and their binding epitopes on HA. As described in several recent reviews [5,6,7,8], there are two main classes of broadly neutralising mAbs, known to bind to highly conserved domains in the head and stalk of HA, respectively.

Besides the above, there is also a third class of mAbs that can neutralise diverse strains within the same subtype, but do not cross-react with other subtypes [9,10]. Interestingly, several mAbs in this class have been documented to bind to the vestigial esterase (VE) subdomain of HA of the H5N1 subtype and they have been shown to bind to multiple clades of H5N1 viruses [11,12,13,14,15]. However, they typically do not bind to other subtypes of IAV, which suggests that the VE subdomain is conserved within a subtype, but is diverse among subtypes. This review summarises the current knowledge on neutralising mAbs binding to this under-studied subdomain in H5N1 HA and the potential role(s) played by this subdomain in viral infection and pathogenesis.

## 2. Discussion

### 2.1. Localisation of VE in the Three-Dimensional (3D) Structure of HA of H5N1

The VE subdomain of IAV was defined based on its structural homology (54%) to a region of the 9-*O*-acetylesterase domain within the HA–esterase fusion glycoprotein (HEF) of influenza C [16]. VE subdomains are found in HA of both influenza A and B viruses, but their functions are not well-defined. The esterase subdomain of the influenza C virus is found in the HEF, and this subdomain is responsible for cleaving the host receptor to facilitate viral budding [17]. However, in influenzas A and B, the VE subdomain does not play this role; a separate neuraminidase protein fulfils the receptor-destroying role of the esterase.

**Figure 1:**
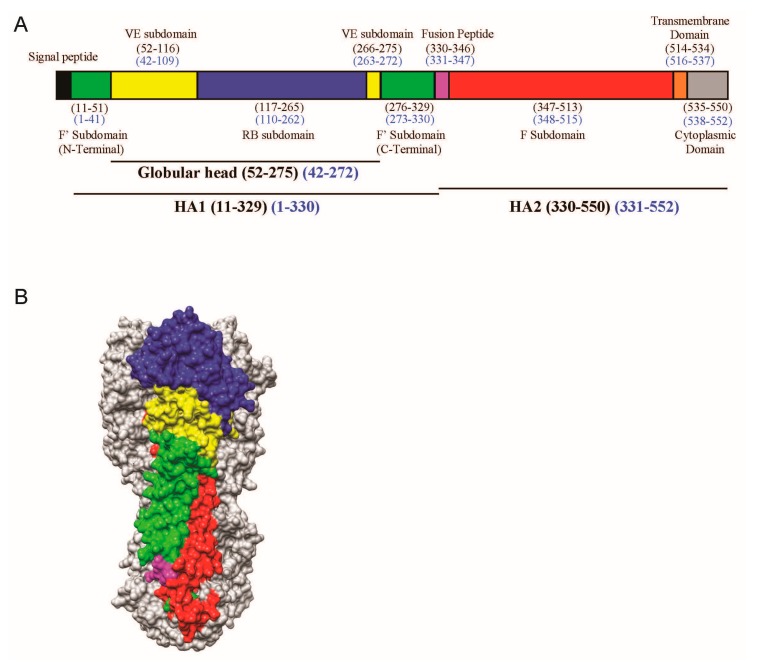
(**A**) Schematic view of the H5 influenza viral haemagglutinin (HA) protein showing the different motifs present in HA. The stalk domain can be further divided into the F’ (green) or F (red) subdomains, whereas the globular head consists of the receptor binding (RB) subdomain (blue) and (vestigial esterase) VE subdomain (yellow), followed by the fusion peptide (magenta), transmembrane domain (orange), and cytoplasmic domain (grey). Numbering is indicated in the H3 (black) and H5 (blue) formats; (**B**) The corresponding segments in 3D are shown as a surface contour representation of one of the protomers of the H5 trimer from A/Vietnam/1203/04 (VN04) (PDB ID: 2FK0).

The amino acid residues in the VE subdomain are highly conserved within a subtype of IAV, but are variable between subtypes [18]. The H3 numbering is used throughout this paper. The VE of H5N1 is made up by two noncontinuous sequences, namely residues 52 to 116 and residues 266 to 275 (Figure 1A). Structurally, VE is located in HA1 and located between the receptor-binding subdomain in HA1 and the membrane-proximal stem made up mainly of HA2 (Figure 1B). Comparison of the crystal structures of HA of the H3, H5, and H9 subtypes did not reveal any dramatic difference in secondary or tertiary structure of the VE subdomains [18].

### 2.2. Generation of Neutralising mAbs Binding to the VE Subdomain of H5N1

Murine mAb 9F4 is a neutralising antibody binding to the VE subdomain of H5N1 [11]. Recombinant HA protein encoded by a clade 1 virus (A/chicken/Hatay/2004 (H5N1)) was expressed in insect cells using baculovirus vectors [19]. The recombinant H5 HA was used to immunise mice, and hybridomas were then generated. mAb 9F4 (IgG2b isotype) was selected based on its ability to prevent multiple H5N1 pseudotyped viral particles from entering host cells, and it was found to neutralise live H5N1 viruses of clade 2.2.2. in vitro and in vivo [20]. The broad cross-neutralising activity of mAb 9F4 was subsequently confirmed by its ability to neutralise newly emerged H5N1 viruses belonging to clades 2.3.4 [21] and 2.3.2.1a [11].

**Figure 2:**
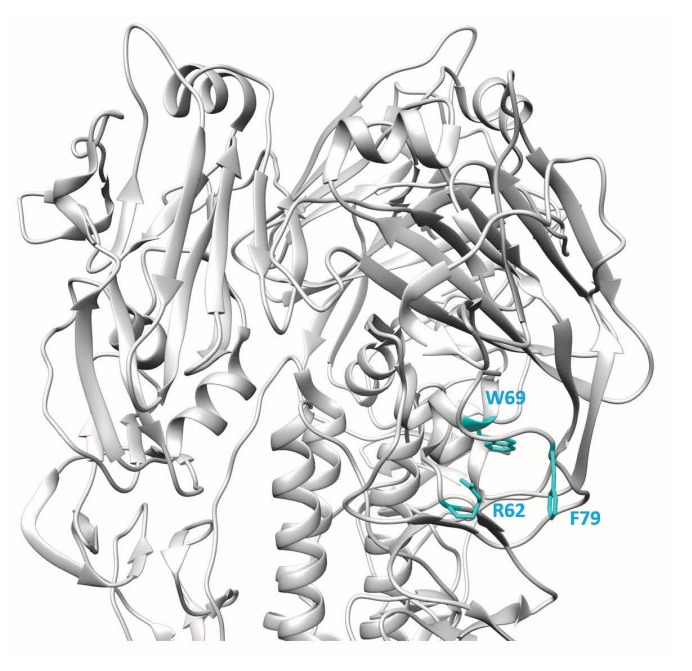
3D ribbon representation showing one of three protomers of the VN04 (PDB ID: 2FK0) H5 trimer. Residues bound by the mAb 9F4 are shown.

Epitope mapping of the mAb 9F4 was guided by analysis of antibody escape mutants and in silico antigenic prediction [11]. Sequencing of the escape mutants showed the presence of two mutations in the *HA* gene: R62 mutated to G and T163 mutated to S. Site-directed mutagenesis and binding assays showed that R62 was the critical residue making contact with mAb 9F4, whereas T163S was a passenger mutation. Other residues in HA that could be involved in the interaction with mAb 9F4 were predicted utilising in silico prediction methods and proximity of the predicted residues to R62. After carrying out binding analysis with the substitution mutants, two additional amino acid residues, namely W69 and F79, were also found to be important for the interaction between HA and mAb 9F4. Thus, the binding of mAb 9F4 requires at least three noncontinuous amino acid residues, namely R62, W69, and F79, which form an epitope in the VE subdomain of HA (Figure 2). Binding and neutralisation studies showed that R62 makes critical contact with mAb 9F4, whereas W69 and F79 may not directly interact with mAb 9F4, but may affect the stability of the conformational epitope in HA.

**Figure 3:**
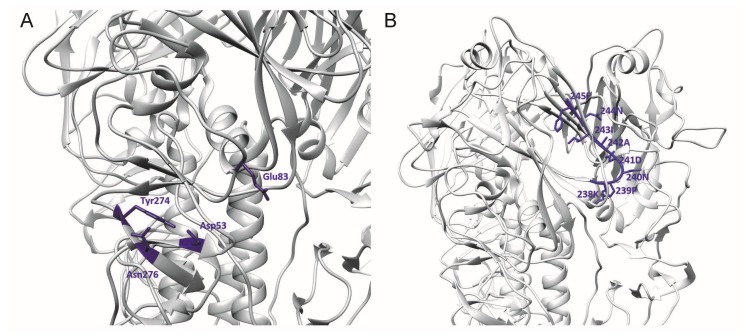
3D ribbon representation showing one of three protomers of the VN04 (PDB ID: 2FK0) H5 trimer. Antibody-binding epitopes of (**A**) the mouse and (**B**) humanised H5M9 are shown.

Previously, two mAbs—H5M9 [14] and 4F5 [15]—were reported to interact with amino acid residues close to the binding site of 9F4. H5M9 was produced by immunising mice with the HA protein of A/goose/Guangdong/1/96 (H5N1). Crystal structures of H5M9 complexed with the HA protein of VN04 (H5N1) revealed that the antibody-binding epitope is located in the VE subdomain and is comprised of amino acids D53, Y274, E83, and N276 (Figure 3A).

Further to the above study, the humanised H5M9 antibody was generated by transferring the mouse complementarity determining region (CDR) residues together with four key framework region (FR) residues onto the FR of the human antibody [22]. Through epitope mapping studies, a linear epitope was identified on the receptor-binding subdomain of HA that was H5N1-specific and conserved. The linear epitope targeted by the CDR-grafted humanised H5M9 antibody was present from amino acid residues 238 to 245 with the sequence “KPNDAINF” (Figure 3B). However, this epitope was different from the reported epitope of mouse mAb H5M9 [14] and is present outside the VE subdomain. The reason for this difference has not been established.

**Figure 4:**
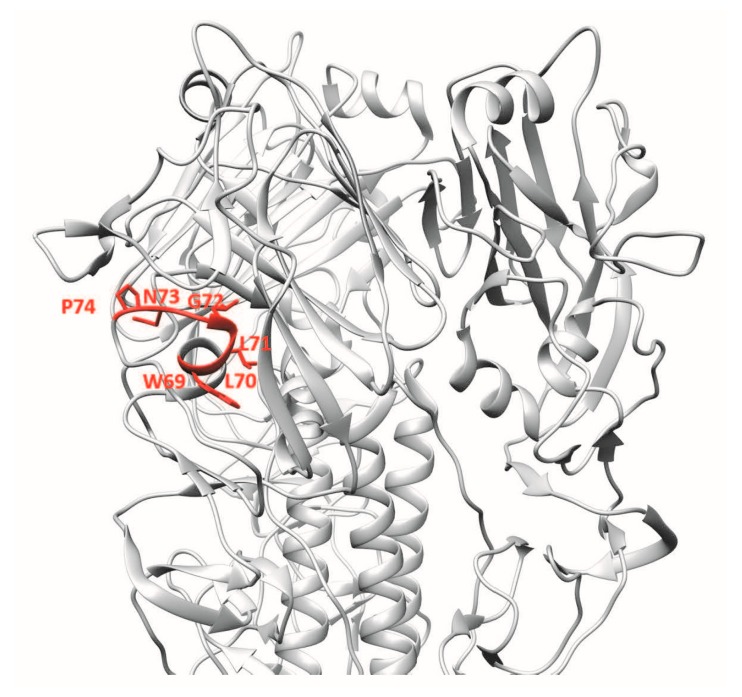
3D ribbon representation showing one of three protomers of the VN04 (PDB ID: 2FK0) H5 trimer. Antibody-binding epitopes of 4F5 are shown.

For the generation of mAb 4F5, a library of phage-displayed human single-chain variable fragments (scFvs) containing 6.0 × 10^8^ antibody clones was generated from lymphocytes of individuals vaccinated with H5N1 vaccine. Using recombinant HA1 protein of H5N1 for screening, the 4F5 scFv was identified as having neutralising activity against H5N1 viruses of clades 2 and 9. mAb 4F5 was reported to bind the 69WLLGNP74 epitope [15] (Figure 4). Significant abrogation of binding in Western blot analysis was observed when WLLGNP was mutated to WRRGNP.

**Figure 5:**
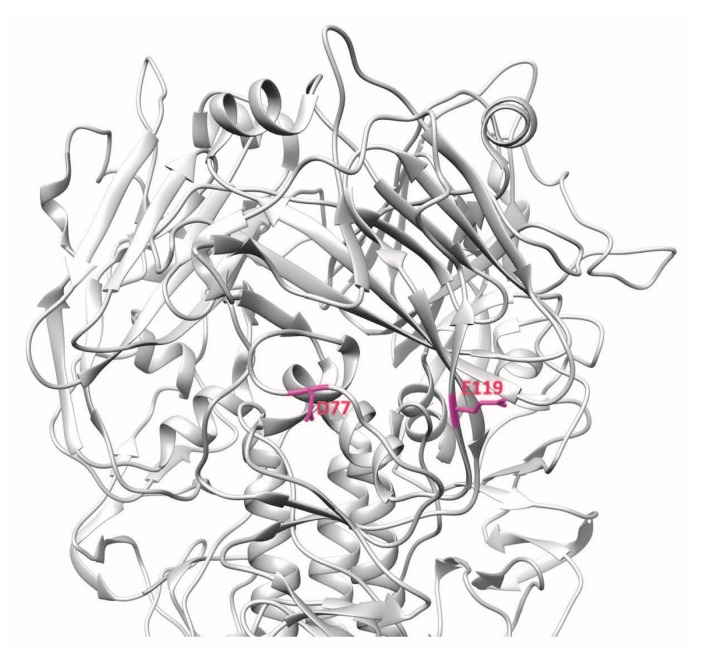
3D ribbon representation showing one of three protomers of the VN04 (PDB ID: 2FK0) H5 trimer. Antibody-binding epitopes of 100F4 are shown.

Another human mAb, 100F4, neutralises multiple clades of H5N1 and binds outside the receptor-binding subdomain of H5N1 HA [12,23]. Memory B cells from peripheral blood mononuclear cells of a patient who recovered from H5N1 infection were immortalised and culture supernatants were screened by HA pseudotype-based neutralisation assay. 100F4-producing hybridoma culture supernatant showed neutralisation activity against HA and neuraminidase pseudotype viruses of H5N1 A/Shenzhen/406H/2006. Hence, after one round of subcloning, total RNA was isolated and *VH*, *Vκ*, and *Vλ* gene segments were amplified by reverse transcription PCR and inserted into *Drosophila* S2 cell expression vectors containing constant regions of human *γ1*, *κ1*, and *λ1* gene segments. Culture supernatants were harvested, and the human monoclonal antibodies in these culture supernatants were purified. 100F4 was reported to bind to amino acid residues at positions D77 and E119, which are found in the VE subdomain of H5N1 HA [12] (Figure 5).

**Figure 6:**
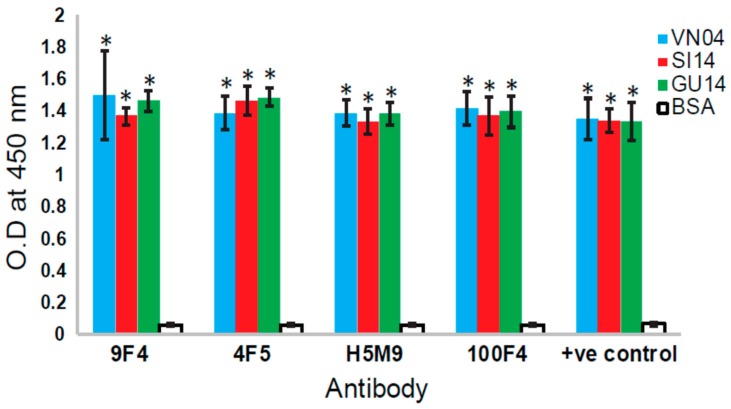
Analysis of the binding of different VE-binding mAbs to H5N6 HA. Ninety-six-well ELISA plates were coated with 100 ng/well of recombinant His-tagged HA protein from A/Vietnam/1203/2004(H5N1)(VN04), A/Guangzhou/39715/2014(H5N6)(GU14), and A/Sichuan/26221/2014(H5N6)(SI14) (purchased from Sino Biological, Beijng, China). Bovine serum albumin (BSA) was coated as negative control. In total, 1.25 µg/mL of each recombinant human and mouse–human chimeric antibodies (produced as described in [11,21]) were added. Anti-His mouse mAb was used as the positive control antibody. After incubation and washing, horseradish peroxidase-conjugated anti-mouse or anti-human IgG were added as secondary antibodies. After incubation and washing, the substrate 3, 3′, 5, 5′-tetramethylbenzidine was added, incubated until optimum development of colour, and stopped with 2 M sulphuric acid. The absorbance at 450 nm was measured. Three independent experiments were performed, and data is shown as the mean of 3 experiments ± SD. Two-tailed Student’s *t*-test was used to evaluate the significance of differences between sample sets. * Indicates statistically significant difference of *p* < 0.05 when compared to BSA.

Since the first human infection of H5N1 IAV in 1997, the virus has evolved and reassorted to give rise to different clades of H5N1 as well as H5Nx viruses. In May 2014, the first human case of avian H5N6 IAV infection was identified in China’s Sichuan Province [24]. As 100F4 has been shown to bind to newly emerged H5N6 viruses [13], we performed ELISA to determine if H5M9, 4F5, or 9F4 bind H5N6 HA. As shown in Figure 6, all these mAbs bound the HA proteins of two human isolates of H5N6.

Although the four mAbs described above bind to the VE subdomain of H5N1 HA, they are binding to different epitopes. Indeed, competitive binding assays showed that the epitope of mAb 9F4 is distinct from those of mAbs H5M9, 4F5, and 100F4 [11]. The presence of multiple neutralisation epitopes in VE suggests that this subdomain could play an important role in viral entry (see Section 2.4).

### 2.3. In Vivo Studies of VE-Binding mAbs

Antibodies function primarily by binding to pathogens and either directly neutralise their ability to infect host cells or stimulate a broad range of Fc-dependent responses such as antibody-dependent cell-mediated cytotoxicity (ADCC), complement-dependent cytotoxicity, or antibody-dependent cellular phagocytosis (see review [25]). Whether a particular antibody will be able to directly neutralise IAV depends largely on the antibody’s binding epitopes. For instance, antibodies that can directly neutralise IAV generally target the globular head within the HA region, prevent viral attachment or membrane fusion, and do not rely on Fc–FcR interactions for protection against the virus [26]. In contrast, antibodies that bind to the stalk region generally rely on Fc-mediated responses to remove IAV or stimulate the destruction of infected cells [26,27]. The ability of antibodies to stimulate Fc-dependent responses depends on the antibody’s isotype, as a particular Fc region can only interact with specific Fc receptors present on immune cells to elicit distinct downstream responses (see reviews [28,29]). To a certain extent, the antibody’s binding epitope can affect its ability to stimulate Fc-dependent responses, presumably due to the accessibility of its Fc region when bound [27]. Furthermore, the binding site of the antibody can subject it to competitive binding with other IAV antibodies in a polyclonal setting and negatively impact its ability to induce ADCC [30].

As discussed above, several H5N1-neutralising antibodies that bind the VE subdomain have been described. Of these, 100F4 was shown to have both prophylactic and therapeutic efficacies which were able to outperform a five-day treatment regime with 10 mg/kg of oseltamivir in mice challenged with H5N6 or H5N8 [13]. To assess the importance of ADCC function for 100F4 protection against influenza, Wang and colleagues generated chimaeric versions of 100F4, either with a mouse IgG2a Fc region which preferentially stimulates ADCC or with a D265A mutation which is unable to stimulate ADCC [31]. When tested in vivo, 100F4/IgG2a, but not 100F4/D265A, was able to confer protection in mice infected with the H5 strains A/Shenzhen/406H/2006, A/chicken/Netherlands/14015526/2014, or A/chicken/Shanxi/2/2006, suggesting that the ADCC pathway was essential for 100F4 protection. In contrast, a D265A mutation did not affect the ability of the HA globular head binding antibody 65C6 to confer protection against all three strains in mice.

Besides mAbs targeting H5N1, several neutralising mAbs for other IAV subtypes have also been found to bind to VE. In a recent study, the human mAb H3v-47 was isolated from experimental vaccine candidates immunised with the swine-origin H3 strain A/Minnesota/11/2010 [32]. Crystal structure analyses revealed that H3v-47 recognises a unique epitope that spans the receptor-binding and VE subdomains. In vitro analyses demonstrated that H3v-47 was capable of binding to and neutralising multiple human and swine strains of H3N2. With an ELISA-based binding and NK cell activation assays, H3v-47 was shown to be able to bind FcγRIIIa and induce ADCC. When tested in vivo, H3v-47 was able to confer protection in mice challenged with the H3 strain A/Minnesota/11/2010. When used in a prophylactic or therapeutic setting, a 1 mg/kg dose of H3v-47 was sufficient to reduce the severity of clinical disease and prevent the fatality of mice due to H3N2 infections.

In studies of H7 viruses, the mouse mAb 1H5 was also shown to bind a broad range of H7 strains, including one North American and all Eurasian strains [33]. Mutations of R57K, a residue within the VE subdomain, resulted in reduced binding of 1H5 to the mutant H7 HA protein, suggesting that 1H5 binds to the VE subdomain within the H7 virus. 1H5 was unable to neutralise H7N9 IAV in vitro, but was able to confer protection when administered either as a prophylactic or therapeutic in A/Shanghai/1/13 (H7N9)-challenged mice. Reporter assays demonstrated that 1H5 was able to activate Fc receptors. To characterise further, the mechanism of 1H5 protection in vivo, chimaeric 1H5/IgG2a and 1H5/IgG2a/D265A antibodies were generated. As expected, 1H5/IgG2a was able to confer protection against A/Shanghai/1/13 challenge in mice at doses as low as 0.3 mg/kg. 1H5/IgG2a/D265A was also able to confer protection in infected mice, albeit only at a higher dose of 1 mg/kg. This is surprising given that 1H5 was not able to neutralise H7N9 IAV directly in vitro, suggesting that the protective effect of 1H5 is in part dependent on its Fc region and that alternative mechanisms may also contribute to the ability of 1H5 to resolve H7 infections [33].

Antibodies targeting the VE subdomain have also been described to be effective against influenza B infections. 46B8 is a human antibody isolated from post-immunisation blood donors. In vitro, 46B8 was shown to be able to neutralise 11 different strains of influenza B virus (IBV) specifically by blocking pH-induced conformational changes of HA, thus preventing membrane fusion. In both NK cell activation and target cell lysis assays, 46B8 was shown to be able to specifically stimulate ADCC. In mice challenged against a minimum lethal dose of IBV, a single intravenous injection at a dose of 15 mg/kg was able to confer protection when administered 24 h, 48 h, or 72 h post-infection. Apart from exceeding the efficacy window of oseltamivir of 48 h post-infection, treatment with 46B8 provided better protective outcomes as compared to a five-day oseltamivir treatment regime in IBV-challenged mice. Interestingly, combination therapy of 46B8 and oseltamivir reduced IBV titres in lungs of infected mice when compared to treatments with 46B8 or oseltamivir alone [34]. Another influenza B-specific mAb, CR8071, also bound to VE and showed neutralising activities in vitro and in vivo [35]. However, in contrast to 46B8, CR8071 did not block pH-induced conformational change of HA.

Although the VE-targeting mAbs 100F4 and 46B8 are specific for different viral types of influenza, many similarities exist between them. When tested in vitro, 100F4 was able to directly neutralise A/Shenzhen/406H/2006 and A/chicken/Netherlands/14015526/2014 [31] by binding to the virus and preventing low-pH-triggered membrane fusion after endocytosis [12]. 46B8 was also able to neutralise IBV via the same mechanism. In contrast, H3v-47 did not prevent low-pH triggered membrane fusion, but instead neutralised IAV by preventing viral egress [32]. All three antibodies have broad neutralising capabilities within specific viral subtypes and are capable of initiating ADCC, possibly due to the localisation of VE in the 3D structure of HA and accessibility of its Fc region when bound. More importantly, 100F4 and 46B8 have been shown to outperform oseltamivir in vivo, demonstrating the utility of VE-targeting antibodies as anti-influenza therapeutics. It will be interesting to see if other antibodies of this class share similar features with 100F4, 46B8, or H3v-47, or how their ADCC-stimulating capabilities may be affected when used in a polyclonal setting.

### 2.4. Role of Residues within VE in Regulating HA Protein Acid Stability

IAV entry into host cells involves attachment to sialic acid-containing receptors, followed by endocytosis of the virus into endosomes where the reduction in pH induces a conformational change in HA, leading to membrane fusion and release of the viral genome into the cytoplasm [2]. The first step is dependent on the receptor subdomain located in HA1 and has been well-characterised and summarised in recent reviews [36,37,38].

In contrast, the subsequent steps involving pH-induced conformational changes in HA followed by membrane fusion appear to be more complex. In particular, the level of acidic pH required to induce the required conformational change in HA varies between different subtypes as well as between different strains of the same subtype (see reviews [39,40]). The avian H5 subtypes of IAV are classified into low-pathogenicity avian influenza virus (LPAIV) and highly pathogenic avian influenza virus (HPAIV) based on their abilities to cause mild and severe disease, respectively, in chickens (*Gallus gallus domesticus*) [41]. Various studies have shown that the fusion of HA of HPAIV H5N1 virus occurs at a higher pH than that of LPAIV [39,40]. Importantly, the pH stability of HA seems to be related to the adaption of H5N1 to host cells as well as viral transmissibility and pathogenesis.

While different residues in H5N1 HA, particularly those near to or in the fusion peptide pocket, have been reported to contribute to its pH stability, it is interesting to note that some residues within the VE subdomain also appear to be directly involved (see review [39]). This is not surprising given that the 110-helix found in VE is involved in the interaction between HA1 and the loop B of HA2, which undergoes a loop-to-helix transition upon protonation at low pH [18]. For example, several residues in HA were found to be responsible for differences in pathogenicity of two H5N1 isolates, namely a HPAIV strain (A/chicken/Hong Kong/YU562/2001) and a moderately pathogenic strain (A/goose/Hong Kong/437-10/1999), and two of these residues are found in the VE subdomain [42,43]. It was further shown that the pH stability of HA protein is correlated with the pathogenesis of H5N1 virus and that the higher activation pH in the HPAIV is mainly due to mutations N104D and T115I, which are located at the *N*- and *C*-termini of the 110-helix, respectively [43]. These mutations are hypothesised to affect the stability of the 110-helix and its interaction with HA2, thus resulting in higher sensitivity to pH-induced conformational change.

Another residue within the 110-helix was also reported to affect the stability of HA. Thus far, highly pathogenic H5N1 has not acquired the ability to transmit efficiently between humans. In a study to understand the mutations required by H5N1 to acquire this ability, Herfst et al. created a mutant virus with three mutations (Q226L and G228S in HA and E627K in PB2) and passaged it serially in ferrets [44]. Interestingly, viruses isolated from six ferrets that acquired the virus via airborne transmission have two additional mutations in HA, namely H110Y and T160A. H110 is found in the middle of the 110-helix in VE, and structural analysis showed that when this is mutated to Y110, it forms a hydrogen bond with the N413 of the adjacent monomer, resulting in an increase in the stability of the HA trimer [45].

## 3. Conclusions

**Figure 7:**
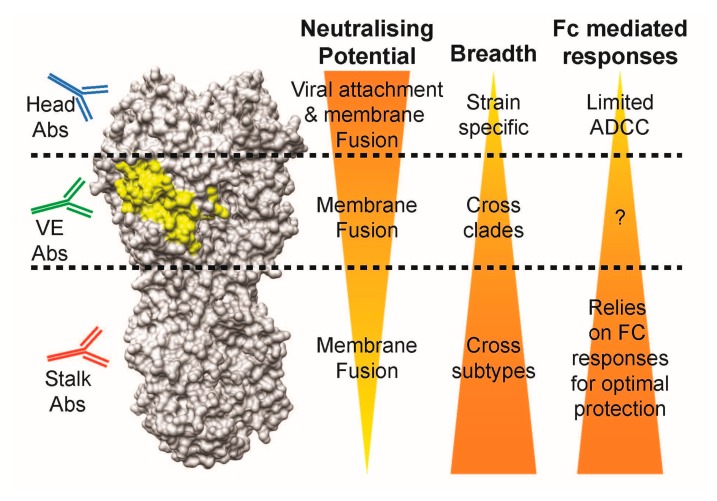
Comparison of antibody characteristics between head-, VE-, and stalk-targeting antibodies. Abs: antibodies, Fc: fragment crystallisable, ADCC: antibody-dependent cell-mediated cytotoxicity.

Anti-head antibodies are potent regarding the direct neutralisation of specific viral strains with limited Fc-mediating potential, while anti-stalk antibodies lose direct neutralisation potential, but gain additional breadth across viral subtypes and Fc-mediated responses in comparison. Taken together with the characteristics of head- and stalk-class antibodies, we speculate that most of the VE-targeting antibodies lie in the intermediacy in terms of neutralising potential and breadth when compared to head and stalk antibodies: (1) they possess direct viral neutralisation capabilities via the inhibition of membrane fusion, but not viral attachment; (2) they are able to protect across different strains within the same influenza subtype; and (3) they are able to stimulate Fc-mediated responses (Figure 7). Whether VE-binding antibodies identified in the future follow this trend remains to be determined.

Given their distinctive properties, the VE-binding anti-H5N1 mAbs described in this study may be considered for usage in combination approaches with head or stalk antibodies to determine if they can act synergistically and increase the potency of therapeutic treatment. Most of them have not been characterised with regards to ADCC, and structural analysis of the antigen–antibody interface is also lacking. It will also be important to use these mAbs to determine the abundance of VE-binding antibodies induced by H5N1 vaccines developed for pandemic preparedness.
